# Transcriptome variations among human embryonic stem cell lines are associated with their differentiation propensity

**DOI:** 10.1371/journal.pone.0192625

**Published:** 2018-02-14

**Authors:** Changbin Sun, Jiawen Zhang, Dongmin Zheng, Jian Wang, Huanming Yang, Xi Zhang

**Affiliations:** 1 BGI Education Center, University of Chinese Academy of Sciences, Shenzhen, China; 2 BGI-Shenzhen, Shenzhen, China; 3 China National GeneBank, BGI-Shenzhen, Shenzhen, China; 4 James D. Watson Institute of Genome Sciences, Hangzhou, China; University of Texas at Austin Dell Medical School, UNITED STATES

## Abstract

Human embryonic stem cells (hESCs) have the potential to form any cell type in the body, making them attractive cell sources in drug screening, regenerative medicine, disease and developmental processes modeling. However, not all hESC lines have the equal potency to generate desired cell types *in vitro*. Significant variations have been observed for the differentiation efficiency of various human ESC lines. The precise underpinning molecular mechanisms are still unclear. In this work, we compared transcriptome variations of four hESC lines H7, HUES1, HUES8 and HUES9. We found that hESC lines have different gene expression profiles, and these differentially expressed genes (DEGs) are significantly enriched in developmental processes, such as ectodermal, mesodermal and endodermal development. The enrichment difference between hESC lines was consistent with its lineage bias. Among these DEGs, some pluripotency factors and genes involved in signaling transduction showed great variations as well. The pleiotropic functions of these genes in controlling hESC identity and early lineage specification, implicated that different hESC lines may utilize distinct balance mechanisms to maintain pluripotent state. When the balance is broken in a certain environment, gene expression variation between them could impact on their different lineage specification behavior.

## Introduction

Human embryonic stem cells (hESCs), derived from inner cell mass (ICM) of human blastocysts [1], have the capacity to differentiate into any functional cell type of the three germ layer (defined as pluripotency), and self-renew indefinitely *in vitro*, making them attractive cell sources in drug screening, regenerative medicine, disease and developmental processes modeling [2–4]. Since the first hESC line established [1], many lines have been cultured from different laboratories in the past two decades around the world [5]. The growth of ES cells as a pluripotent population requires a balance between survival, proliferation, and self-renewal signals, thus understanding the molecular mechanism involved in self-renewal and pluripotency of embryonic stem cells are critical for better culture method establishment and further application [6]. So far, considerable efforts have already been made to explore the precise molecular mechanisms of regulating pluripotency and self-renewal in embryonic stem cells [7–14]. Several genetic regulators play pivotal roles in identity control of hESCs have been identified, including extracellular signaling factors [7, 12, 14], transcription factors[8, 9], cell cycle regulators [12], microRNA [13, 15], genes involved in maintaining chromosomal stability [10], and DNA methylation [16]. Several cocktails of these regulators have been successful to reprogram somatic cells into induced pluripotent stem cells (iPSCs) [17, 18]. These regulators precisely form a complex circuit that represses genes required for differentiation and holds the ESCs in a pluripotent state [6, 19]. However, not all hESC lines are equal in their potency to differentiate into desired cell types *in vitro*, significant variations have been observed in the differentiation efficiency of various human ES cell lines. The precise underpinning molecular mechanisms are still largely unclear [20–24].

Several studies have been reported that hESC lines differ in the ability to differentiate into distinct cell types while they can commonly maintain their pluripotent state in culture [20–23]. Osafune, et al characterized 17 hESC lines differentiation potential *in vitro* by assessing the expression of genes that are the markers of the three germ layers and their derivatives at four time points during spontaneous or directed differentiation. They demonstrated that hESC lines have different propensity to differentiate into certain lineages or cell types [20]. Bock, et. al. established genome-wide reference maps of DNA methylation and gene expression of 20 previously derived human ES lines and 12 human iPS cell lines, and assessed their differentiation propensity *in vitro* [21]. In addition, WNT3 and miR-371-3 have been identified as biomarkers that are capable of predicting the definitive endoderm and neural differentiation propensity of human pluripotent stem cells, respectively [22, 23]. All these studies indicated that different hESC lines are distinct in their ability to form certain types of cells, although they have the common defined characteristics of self-renewal and pluripotency. Genetic and epigenetic variations may contribute to functional variability between cell lines. However, how these variations ‘lock’ the pluripotent state and differentially respond to development signaling that lead to differentiation bias remain to be elucidated. Understanding the mechanisms will facilitate finding appropriate culture conditions to overcome the propensity and establish more efficient differentiation protocol.

Several studies have already explored the gene expression profiles of hESCs by different techniques [25–28]. Most of them focused on key genes that regulate pluripotency and maintain the undifferentiated state [24]. Some markers have been identified to predict certain cell type differentiation propensity in human pluripotent stem cell [22, 23]. However, there were hundreds even thousands of genes show different expression between cell lines. Whether these genes are associated with differentiation bias or they collectively influence hESCs differentiation behavior have not been investigated so far.

In this work, we wanted to find out whether transcriptome variations among hESC lines were associated with developmental processes that may eventually affect hESCs differentiation behavior. We compared transcriptome variations of four hESC lines H7, HUES1, HUES8 and HUES9 by RNA-Seq. We totally identified 19,429 expressed genes, in which 3,571 genes, including 335 transcription factors (TFs), were differently expressed at least between two lines. Gene Ontology (GO) functional annotation demonstrated that these differentially expressed genes are significantly enriched in developmental processes, such as ectoderm, mesoderm and endoderm development. These functional enrichments of DEGs were shown to be associated with differentiation propensity and were in line with lineage bias *in vitro*. Among these DEGs, pluripotency factors, such as POU5F1 and NANOG, and genes involved in signaling pathways transduction, such as BMP, WNT, FGF and FZD family genes, also showed significantly different expression level among the four lines. These genes not only play key role in hESC identity controlling but also influence early lineage specification. Therefore, these data implicated that different hESC lines, which showed distinct differentiation propensity, utilized different balance networks to maintain pluripotent state. When the balance is broken in a certain environment, gene expression variation between them could impact on their different lineage specification behavior.

## Materials and methods

### Cell culture

The Ethics Committee of BGI-IRB approved this study. H7 were obtained from GE healthcare and HUES1, HUES8, HUES9 lines were bought from Harvard University. All hESC lines with passage number between 30 and 40 were used in this study and cultured according to the protocol established in our lab. Briefly, cells were grown in hESC medium containing DMEM/F12 basic medium (Life Technologies), 20% knockout serum replacement (KSR, Life Technologies), 1×L-glutamine (Life Technologies), 1×MEM NEAA (Life Technologies), 0.1 mM 2-Mercaptoethanol (Life Technologies) and 50 ng/mL human FGF2 (Life Technologies) on Mitomycin C (Sigma) treated murine embryonic fibroblasts (MEFs), medium was changed every day. About 7 days, cells were dispersed into small clumps with 1 mg/mL Collagenase IV (Life Technologies) for 20 min at 37°C-, then plated onto Matrigel hESC-qualified Matrix (Corning)-coated dishes in mTeSR^TM^1 medium (Stemcell Technologies) at a ratio of 1:3 to 1:6. In the feeder-free medium, ReLeSR^TM^ (Stemcell Technologies) were used for dissociation and passaging according to the manual.

### RNA-Seq library construction and sequencing

When hESC colonies reached about 80%-90% confluence in mTeSR^TM^1 medium, cells were collected by ReLeSR ^TM^ according to the protocol. Briefly, cells were washed with 1 × DPBS twice and added to appropriate ReLeSR^TM^ for 3–5 min at room temperature, then the appropriate mTeSR^TM^1 medium was added to the cells and shook mildly. Cells were spined at 1,200 rpm and collected in 15mL tubes. All protocols for Illumina sequence preparation, sequencing, and quality control were provided by BGI. Briefly, cells were mixed with TRIzol (Invitrogen) and dissolved for 5 min, then spined at 12,000 × g for 5 min at 4°C. Chloroform was added to the supernate and mixed, then spined at 12,000 × g for 10 min at 4°C. Chloroform/ isopropanol (24:1) was added to the supernate and spined at 12,000 × g for 10 min at 4°C again. The same volume of isopropanol was added to the supernate and stored at -20°C for 1 hr, then spined at 13,600 × g for 20 min at 4°C. Sediment was washed by 75% alcohol and spined at 13,600×g for 20 min at 4°C twice and RNA was dissolved by Nuclease-free water. The purity, integrity, and density of RNA were detected by Nanodrop, Agarose gel electrophoresis and Agilent 2100 BioanaLyzer respectively, then cDNA was synthesized and PCR was used to construct RNA-Seq library. RNA-Seq was conducted by Illumina Hiseq 2000.

### RNA-Seq data processing and differential expression analysis

Reads were mapped to the human genome (GRCh37/hg19) using HISAT2 with default parameters as described in detail in [29]. Raw counts of sequencing reads for the feature of genes were extracted by featureCounts included in the SourceForge Subread package [30].

To identify differential expressed genes, edgeR in the R package was used to import, organize, filter and normalize the data [31]. Expressed genes were selected as their counts per million (CPM) value not less than 1 in at least two samples across the entire experiment while lowly expressed genes were removed for the flowing analyses. Quasi-likelihood F-tests (ANOVA-like analysis) were achieved to identify DEGs according to description in detail in [32]. Genes with fold change (FC) more than 2 and false discovery rate (FDR) less than 0.01 were assigned as DEGs.

SRA files of H1 (GEO accession number: GSM915328 and GSM915329) and HUES64 (GEO accession number: GSM1112834 and GSM1112837) RNA-Seq data were downloaded from https://www.ncbi.nlm.nih.gov/sra. Data analyses of these two cell lines were performed according to the protocol described in [33]. Genes withFC more than 2 and adj.P.Val less than 0.01 were assigned as DEGs.

### GO enrichment analysis

GO and GO-slim enrichment analyses were performed using PANTHER™ Version 13.0 according to [34] with Binomial test type. P-value less than 0.05 were assigned as significance.

### Embryoid bodies (EBs) formation

When hESC colonies reached about 80%-90% confluence in mTeSR^TM^1 medium, cells were dispersed into small clumps with ReLeSR^TM^ according to the manual and transferred to ULA-flasks (Corning) in EBs medium containing DMEM/F12 basic medium, 20% KSR, 1×L-glutamine, 1×MEM NEAA and 0.1mM 2-Mercaptoethanol, the medium was changed every two days.

### Real-time quantitative reverse transcriptase-polymerase chain reaction (RT-PCR)

Total RNA was extracted using TRIzol reagent (Invitrogen) cDNA was transcribed from 300ng RNA for one reaction using PrimeScript RT reagent Kit (TAKARA) according to manufacturer’s protocol. Gene expression of SOX2 (Forward: 5’-AGGATAAGTACACGCTGCCC-3’; Reverse: 5’-TAACTGTCCATGCGCTGGT T-3’), POU5F1 (Forward: 5’-CTTGCTGCAGAAGTGGGTGGAGGAA-3’; Reverse: 5’-CTGCAGTGTGGGTTTCGGGCA-3’), NANOG (Forward: 5’-AATACCTCAGC CTCCAGCAGATG-3’; Reverse: 5’-TGCGTCACACCATTGCTATTCTTC-3’), PAX6 (Forward: 5'-AACAGACACAGCCCTCACAAACA-3', Reverse: 5'-CGGG AACTTGAACTGGAACTGAC-3'), and Nestin (Forward: 5'-GACCCTGAAGGGCA ATCACA-3', Reverse: 5'-GGCCACATCATCTTCCACCA-3') were normalized to inner reference GAPDH (Forward:5’-CCACCAGCCCCAGCAAGAGC-3; Reverse:5’-CAAGGTGCGGCTCCCTAGGC-3’). Data were representative of three independent experiments.

### Neural and cardiac differentiation

For neural progenitor cells (NPCs) differentiation, a modified protocol from [35] were used here. Briefly, when hESC colonies reached about 70%-80% confluence in mTeSRTM1 medium, cells were treated with 0.5mM EDTA in PBS at room temperature for 6 to 10 min and resuspended in NPC differentiation medium supplemented with 10 μM Y-27632 (Sigma). Resuspended cells were then seeded onto fresh matrigel coated plates at densities about 2 × 10^4^ cells/cm^2^ in NPC differentiation medium supplemented with 10 μM Y-27632 for 24 h. Then cells were culture in NPC differentiation medium without Y-27632 and media was changed every day. Cells were harvested at the end of day 7. The NPC differentiation medium consists of DMEM/F12 basic medium, with 20% KSR, 1% NEAA, 1% Glutamax, 10 mM SB431542 and 100 ng/ml Noggin.

For cardiac differentiation, a modified protocol from [36] and [37] was employed. Briefly, when hESC colonies reached about 70%-80% confluence in mTeSR^TM^1 medium, cells were dissociated into single cells with Accutase (Thermo Fisher Scientific) at 37°C for 10 min and then were seeded onto fresh matrigel coated plates at densities 1×10^5^ cells/cm^2^ in mTeSR^TM^1 supplemented with 10 μM Y-27632 for 24 h. Cells then were cultured in mTeSR^TM^1, which was changed daily. When cells reached about 70%-80% confluence (2 days), cells were treated with 6 μM CHIR99021 (Sigma) in RPMI/B27-insulin (Thermo Fisher Scientific) for 48 h (day 0 to day 2). At day 3, The medium was changed to RPMI/B27-insulin with 5 μM IWP2 (Sigma) for another 2 days. At day 5, the medium was changed to RPMI/B27-insulin. At day 7, the cells were transferred to RPMI/B27, and medium was changed every 2 days. At day 15, cells were collected for following analysis.

### Flow cytometry

Cells were dissociated into single cells with Accutase and filtered by 40 μm cell strainers (BD Falcon). Filtered cells fixed with 1% (vol/vol) paraformaldehyde for 10 min and permeabilized with 70% methanol for 10 min at room temperature. For NPCs, cells were stained using 1: 200 rabbit polyclonal IgG PAX6 (ab5790, Abcam) as primary antibody for 1 hour and 1: 1,000 Alexa Fluor 488 goat anti-rabbit IgG H&L (ab150077, Abcam) as secondary antibody for 30 min at room temperature. Rabbit polyclonal IgG were employed as an isotype control. For cardiomyocyte, cells were stained with PE mouse anti-cardiac Troponin T type 2 (TNNT2, 564767, BD Bioscience) for 1 hour at room temperature and PE mouse IgG1 kappa (554680, BD Bioscience) was used as isotype control. All samples were run on BD FACSJazz.

## Results

### Highly expressed genes in hESCs

**Fig 1:**
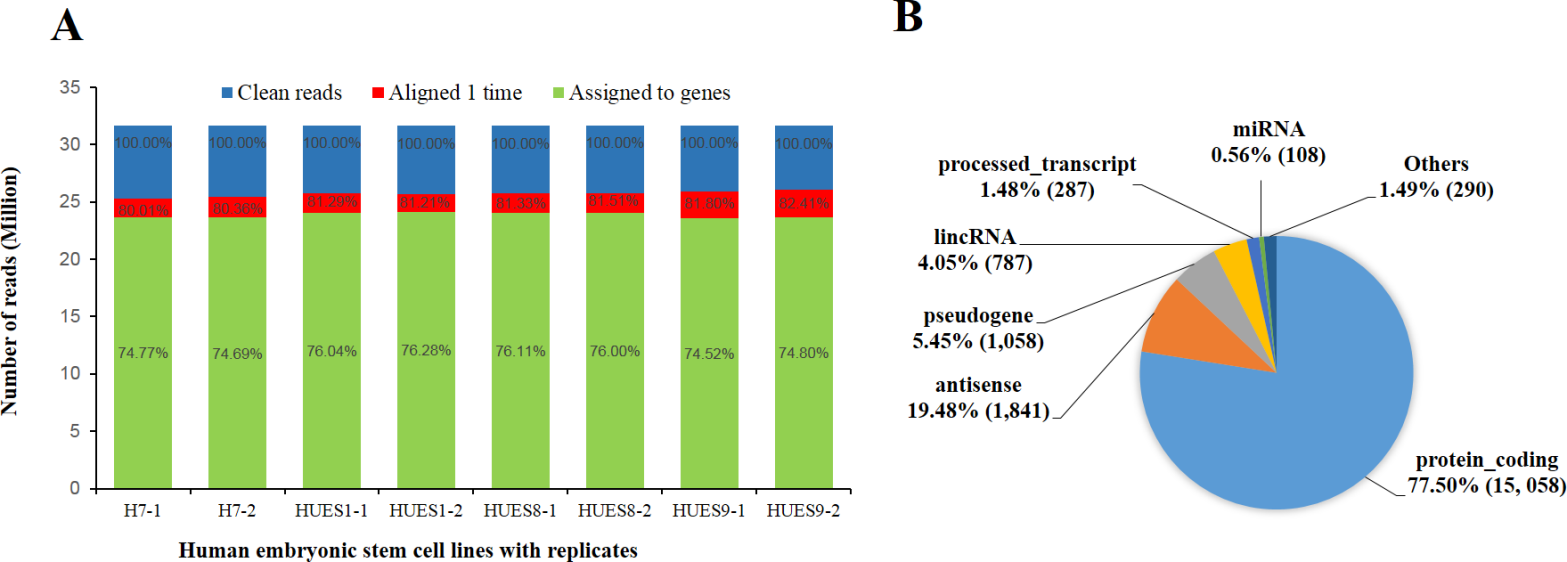
Results of reads mapping to genes expressed in hESC lines H7, HUES1, HUES8 and HUES9. (A) A histogram showing the number of reads uniquely aligned to GRCh37/hg19 genome and assigned to transcriptome for each line with replicates. (B) A pie chart depicting percentage of distinct bio-types annotated in the total genes expressed in hESC lines H7, HUES1, HUES8 and HUES9 after filtering out lowly expressed genes. Number in brackets represent amount of genes in the biotype. CPM of a gene in two or more libraries are larger than 1 considered as expressed genes, otherwise as lowly expressed genes filtered out.

Many factors influence genes expression, such as feeder cells, culture media, additives and passage methods [38]. In order to minimize influence from environmental factors, we synchronously cultured the four hESC lines and passaged three times on the same feeder-free medium before cell collecting for RNA extraction. To remove possible differentiated cells on the clone border, we used ReLeSR™ for dissociation and passaging, which is an enzyme-free reagent without the need for manual removal of differentiated cells. In consideration of biological variability, we pooled three biology samples as one replicate, each line had two independent replicates for library construction and high-throughput RNA sequencing. After adaptor trimming and low-quality filtering, we total obtained more than 253 million clean reads, and each cell line got more than 31 million reads per replicate (Fig 1A). Mapping to human genomes (GRCh37/hg19), more than 80% reads aligned 1 time and about 75% reads uniquely assigned to reference transcriptome. Total 19,429 expressed genes were obtained from following RNA-seq analysis, including 15,058 protein codings, 1, 841antisenses, 1,058 pseudogenes, 787 long intergenic noncoding RNAs (lincRNAs), 287 processed transcripts and 108 micro RNAs (miRNAs) (Fig 1B and S1 Table).

**Fig 2:**
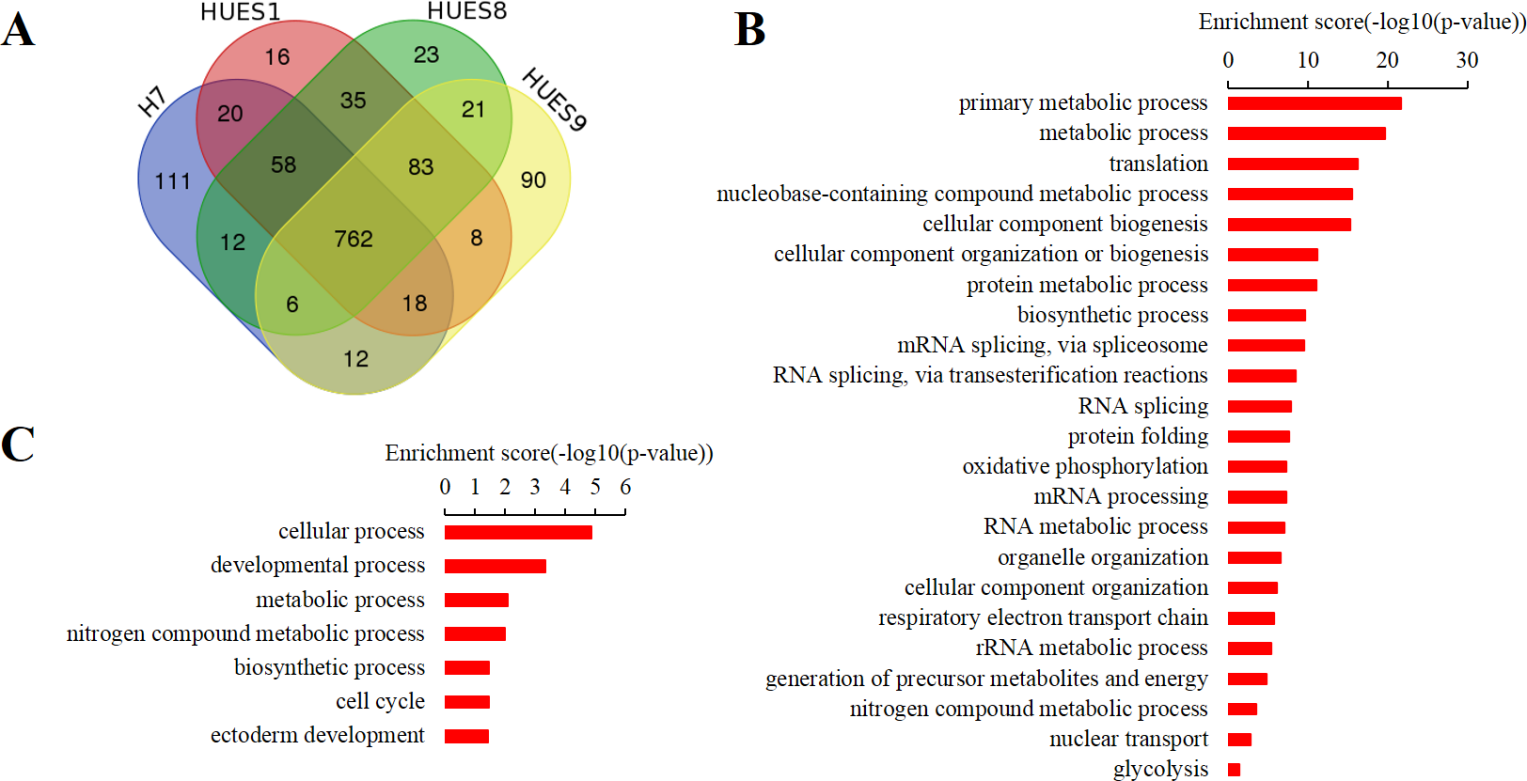
Highly expressed genes in hESC lines H7, HUES1, HUES8 and HUES9. (A) Venn diagram showing top 1000 highly expressed genes ranked by expression level in the four cell lines. Expression level were normalized by transcripts per million (TPM). (B) GO-slim biological process enrichment analysis for 762 commonly high expressed genes shared in the four cell lines. (C) GO-slim biological process enrichment analysis for non-shared high expressed genes in the four cell lines.

Next, we analyzed highly expressed and lineage-specific genes across the four cell lines. Normally, most highly expressed genes regulating pluripotency and self-renewal should be common in all hESC lines while lineage-specific genes should be expressed at a much lower level. Here, we used transcripts per million (TPM) [39] to normalize for sequencing depth and gene length for relative quantity, and then ranked genes abundance according to TPM values. According to the expression level, we assigned the top 1000 ranked genes, accounting for 5.15% of total expressed genes, as highly expressed genes. We combined the top 1000 highly expressed genes from the four cell lines and got 1,275 genes in total (S2 Table), of which 762 genes were shared in the four cell lines (Fig 2A). GO-slim enrichment analysis showed that these shared genes significantly enriched in ribosome and cytosol cellular component that are involved in translation, biosynthetic process, protein metabolic process, mitosis, oxidative phosphorylation biological process. These results were in line with the discovery from previous transcriptome profile studies [25, 27, 28] (Fig 2B). In addition, some pluripotent factors such as POU5F1 (also known as OCT4) and SOX2, were highly expressed in all the four cell lines [19, 27]. In terms of lineage-specific genes, we found these genes are lowly expressed or not detected as expected, and their expression level and ranking are much lower than hESC markers expressed in the four lines (Table 1).

**Table 1:** TPM value and ranking level of lineage-specific genes in hESC lines H7, HUES1, HUES8 and HUES9.

Lineage	Marker	TPM				Ranking			
		H7	HUES1	HUES8	HUES9	H7	HUES1	HUES8	HUES9
hESCs	POU5F1	861.66	414.30	460.03	176.94	86	256	219	826
	SOX2	317.49	654.37	634.96	433.28	347	134	135	228
	PODXL	1322.01	1115.44	1087.66	898.01	49	55	61	75
	CDH1	116.19	183.08	226.13	131.61	1362	779	593	1232
	THY1	383.97	107.21	104.67	71.72	260	1605	1677	2756
	EPCAM	171.07	212.11	199.24	119.70	811	653	717	1416
	CD9	116.48	52.72	53.88	27.92	1356	3792	3735	7452
	ITGB1	131.23	120.19	113.06	120.75	1159	1390	1523	1397
	CD59	33.52	31.81	32.66	25.71	5628	6275	6192	7923
	PROM1	31.34	44.75	44.14	38.73	5977	4514	4638	5591
	PREX1	38.68	28.22	28.71	72.21	4927	6938	6848	2733
	SALL4	142.81	273.96	284.41	305.08	1033	436	424	366
	MYC	67.37	45.14	48.58	18.39	2701	4466	4198	9807
	TDGF1	157.48	116.31	131.87	48.52	907	1455	1212	4388
	DNMT3B	640.81	715.49	888.24	533.03	131	112	82	164
	ALPL	266.77	407.57	369.04	336.52	436	261	307	325
	TDGF1	157.48	116.31	131.87	48.52	907	1455	1212	4388
Mesendoderm	MIXL1	8.18	3.22	2.17	1.31	12617	16066	17066	18297
	GSC	3.55	2.36	3.34	2.05	15474	16882	15976	17616
Mesoderm	T	2.10	2.29	0.87	1.30	16832	16951	18558	18306
	CD34	0.52	0.90	1.68	1.40	18707	18509	17601	18198
	GATA4	0.70	0.84	2.31	0.63	18476	18570	16925	18951
	HAND1	0.18	1.49	4.68	4.38	19186	17827	15010	15858
	TWIST2	5.81	3.08	1.12	4.27	13958	16202	18249	15932
	GATA6	1.04	1.25	2.33	0.83	18041	18099	16898	18724
Endoderm	CXCR4	5.28	10.20	11.27	29.13	14254	12112	11620	7204
	SOX17	1.73	1.74	3.25	1.22	17196	17545	16063	18371
	FOXA2	1.72	1.07	2.90	0.72	17205	18304	16363	18853
	AFP	0.36	0.32	3.48	0.71	18930	19219	15863	18872
	AMN	20.35	19.57	19.89	21.70	8277	8869	8788	8880
	SOX7	10.48	9.34	10.41	7.75	11547	12483	11968	13900
	HNF4A	0.29	0.20	0.85	0.20	19036	19301	18583	19288
	FOXA1	-	-	-	-	-	-	-	-
Ectoderm	PAX6	0.99	13.92	19.80	40.43	18102	10658	8812	5356
	TUBB3	36.09	32.00	24.20	24.45	5271	6235	7768	8203
	SOX1	3.42	7.46	4.60	8.28	15597	13391	15063	13638
	FGF5	-	-	-	-	-	-	-	-

Meanwhile, we also did GO-slim enrichment analyses on those highly expressed genes not shared among the hESC lines to see their functional enrichment. Interestingly, these genes were significantly enriched in developmental processes, especially in ectoderm development (Fig 2C). Among these genes, GLI3 [40–42], ZIC3 [43–45], OTX2 [46–48], CDK4 [49–51], LHX5 [52], HES3 [53] etc., had been reported in several studies and play important role in nervous system development (S2 Table). Next, we analyzed expression variations among the four cells and wanted to investigate its potential impacts on hESCs differentiation.

### Transcriptional variability among hESC lines

**Fig 3:**
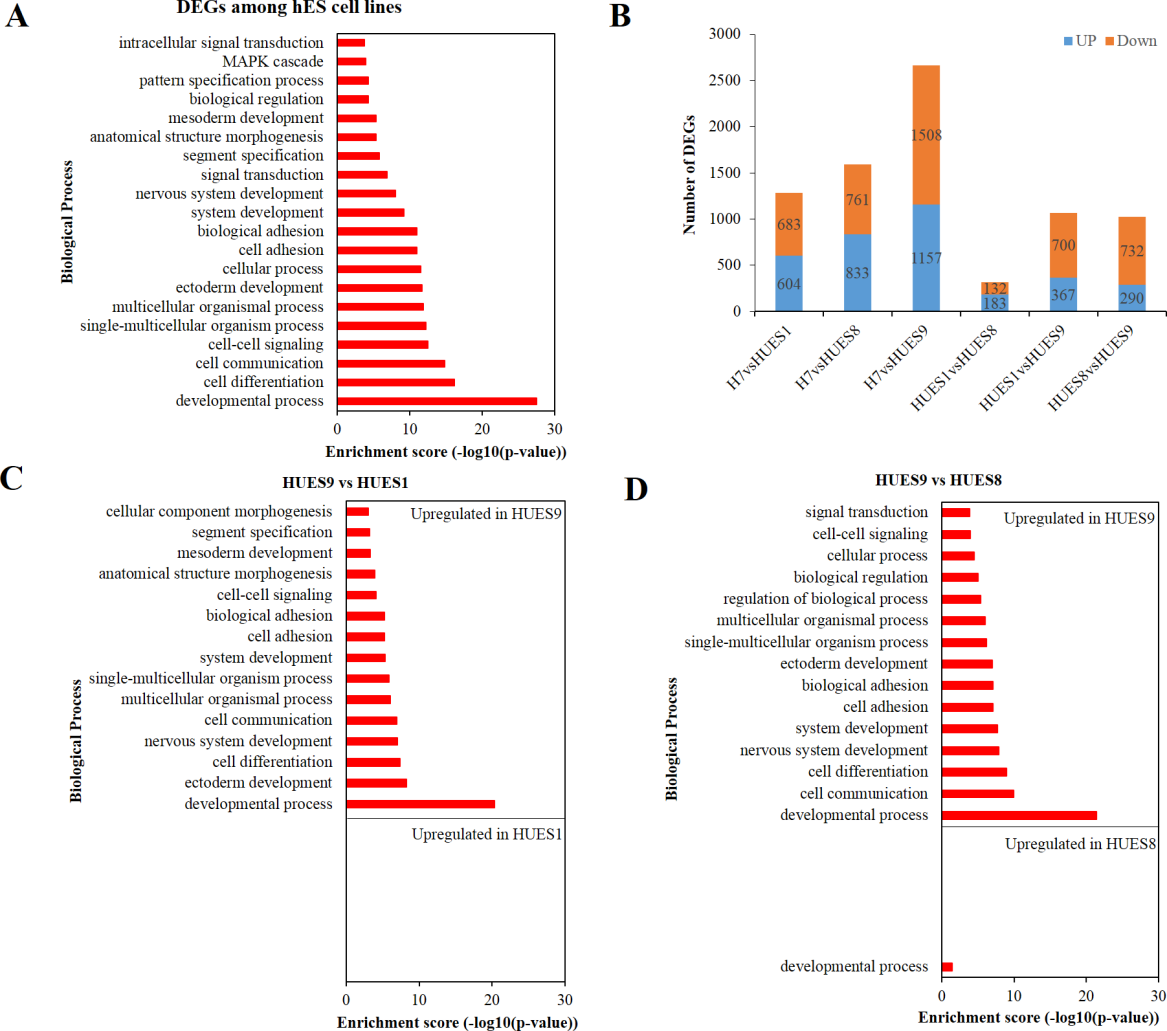
Transcriptional variability among hESC lines H7, HUES1, HUES8 and HUES9. (A) GO-slim biological process enrichment analysis of DEGs at least between two lines. Result shows that these global DEGs are significant enriched in developmental process. (B) Histogram depicting number of DEGs between any two cell lines. HUES1 and HUES8 have the least DEGs compared to other two-two compares. (C) GO-slim biological process enrichment analysis of DEGs between HUES9 and HUES1. (D) GO-slim biological process enrichment analysis of DEGs between HUES9 and HUES8. Although HUES1 is female while HUES8 is male, their expression profiles are more similar than other cell lines. GO-slim enrichment analysis indicates that their DEGs show no significant enrichment in biological process. Compared to HUES9, upregulated genes (logFC > 1 and FDR < 0.01) in these two cell lines exhibit significant enrichment in endoderm development (Note: HUES1 and HUES8 refer to GO complete biological process data, S7 Table) while upregulated genes of HUES9 significantly enriched in nervous system development and ectoderm development. Other results of GO-slim biological process enrichment analysis please see S2 Fig.

Genes with more than 2-fold change (maximum CPM divide by minimum CPM among the four cell lines for each gene) and FDR less than 0.01 were assigned as DEGs. Overall, we identified 3571 DEGs, accounting for 18.38% of the total expressed genes (S3 Table). To investigate whether these DEGs are linked to differentiation propensity variability, we analyzed their functional enrichment. Results of overrepresentation test showed that these DEGs were significantly enriched in nervous system development, ectoderm development, mesoderm development etc. (Fig 3A).

In order to inspect how expression variations associated with differentiation bias, we performed two-two comparison analysis (S4 Table). Results showed that the number of DEGs between HUES1 and HUES8 were the least compared to others (Fig 2B and S3 Fig). GO enrichment analysis of these DEGs showed no significant enrichment in the biological process. On the other side, DEGs between HUES1 or HUES8 and H7 or HUES9, or between H7 and HUES9, were significantly enriched in developmental processes, involving ectodermal, mesodermal, endodermal, nervous system, muscle organ, skeletal system development (Fig 3C and 3D). Previous study had shown that HUES1, HUES8 and HUES9 had various differentiation propensity, with HUES1 inclining toward mesoderm, HUES8 toward mesoderm and endoderm, while HUES9 toward ectoderm [20]. Here, GO-slim enrichment analyses demonstrated that upregulated genes in HUES9 were overrepresented in ectoderm process, while upregulated genes in HUES1 or HUES8 were significantly enriched in endoderm development, implicating that gene expression variability could bring about different hESC lines with distinct differentiation bias (Fig 3C and 3D).

To verify our results, we downloaded public available hESC lines H1 and HUES64 RNA-Seq data to carry out differential expression analysis (S4 Table). Markedly, results showed that genes differentially expressed were enriched in developmental process as well (Figure D in S2 Fig). Together, these results suggest that transcriptional variations between hESC lines are enriched in developmental processes, which could influence their differentiation propensity *in vitro*.

### Expression variability of transcription factors among hESC lines

Transcription factors play key role in genes expression regulation and several master transcription factors can control cell fate decision, such as combination of transcription factors POU5F1, SOX2, KLF4, and C-MYC or OCT4, SOX2, NANOG and LIN28 have been used to transform human fibroblasts into induced pluripotent stem cells (iPSCs) [17, 18]. In the total 19, 429 expressed genes, we assigned 1, 285 genes as TFs according to human transcription factors list downloaded from animal TFDB^2.0^ [54] (S5 Table).

**Fig 4:**
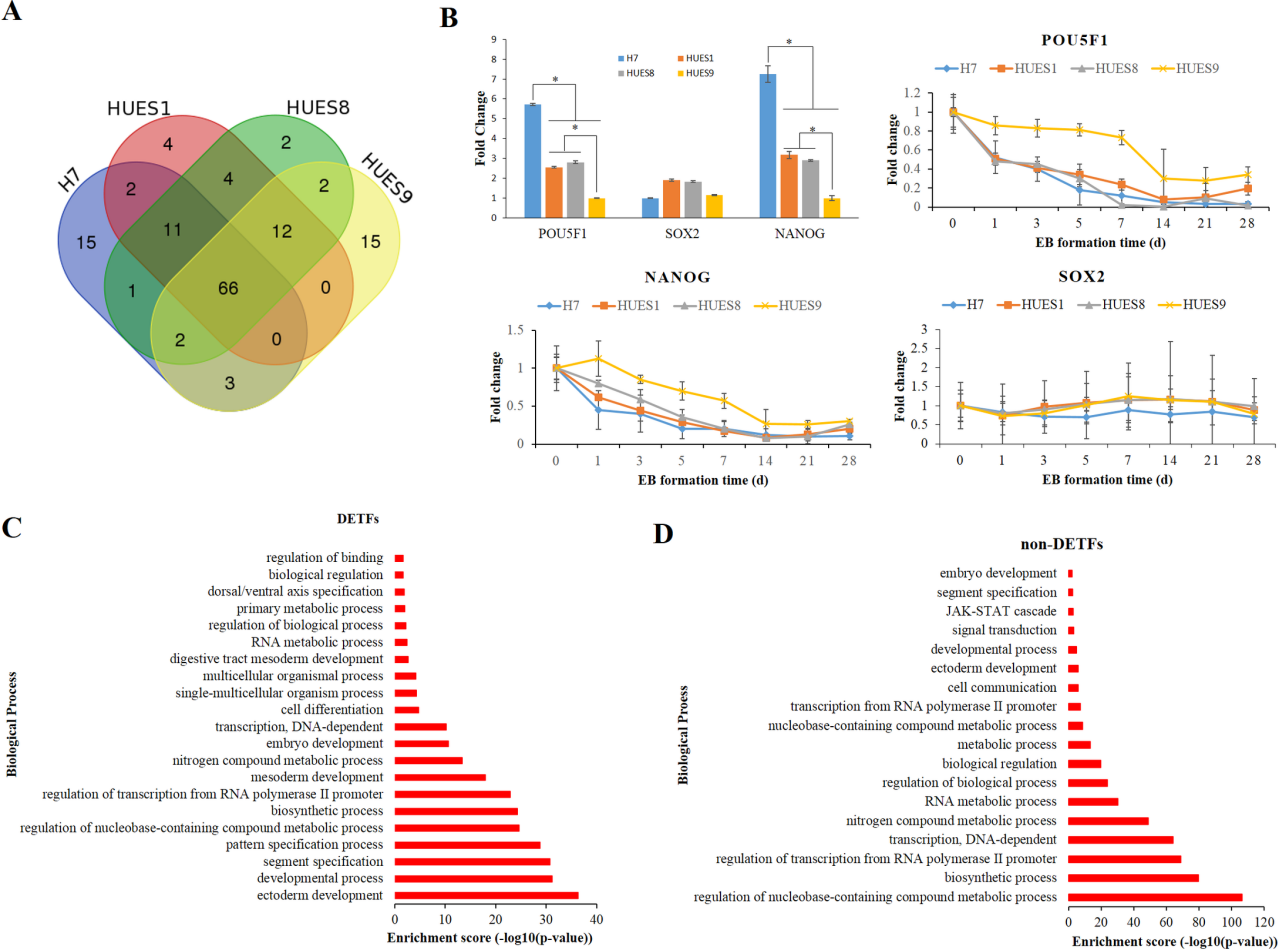
Expression variability of transcription factors among hESC lines H7, HUES1, HUES8 and HUES9. (A) Venn diagram showing top 100 highly expression transcription factors (TFs) TFs in the four cell lines. (B) Expression variation of pluripotent factors POU5F1, NANOG and SOX2 in the four cell lines. Top left histogram exhibits fold change (FC) compared to lowest expressed cell lines (data from RNA-seq). * represents FC >2 and FDR < 0.01. The other three charts show POU5F1, NANOG and SOX2 expression change during EB formation (data from RT-PCR) and fold change of each gene was normalized by expression level of hESC for each line. (C) GO-slim biological process enrichment analysis of DETFs at least between any two cell lines. (D) GO-slim biological process enrichment analysis of non-DETFs among the four cell lines.

Next, we selected top 100 highly expressed transcription factors by their TMP value to see expression variations. We combined the top 100 highly expressed TFs and 74 are shared in all four lines (Fig 4A and S5 Table), including SOX2 and POU5F1 which are well-known auto-regulated TFs in the core transcriptional regulatory circuitry in human embryonic stem cells and contribute to pluripotency and self-renewal [8, 19]. In addition, several TFs that are important regulators of embryonic stem cells pluripotency and self-renewal, such as TCF3 [55], ZSCAN10 [56], SALL4 [57, 58], LIN28A [18], HMGA1 [59], Zic3 [60] and Parp1[61] etc., were in the top 100 TFs set as well. Some of them had been used to generate iPSCs or improve reprogramming efficiency.

Meanwhile, we looked at the number of differentially expressed TFs (DETFs) and their related functions in all the TFs set, 335 DETFs were identified using the same selecting method mentioned above, accounting for 26.07% of the total expressed TFs (S5 Table). Notably, significant difference in POU5F1 and NANOG expression was observed among the four cell lines, which also had been described in previous studies [62, 63]. On the other side, the expression level of SOX2 were only slightly different in the four cell lines. During the EBs formation, the expression changed pattern were similar (Fig 4B). These pluripotent factors also play pivotal role in embryonic stem cell specification to certain germ layer reported in several studies [64, 65]. Therefore, we analyzed biological process enrichment of these DETFs, and compared to non-DETFs to see their difference. Results from GO-slim biological process enrichment analysis showed that these DETFs were significantly enriched in developmental process as well, including ectoderm development, segment specification, mesoderm development and so on (Fig 4C). Differentially, non-DETFs in the four cell lines were mainly involved in metabolic process and biosynthesis process (Fig 4D), which were important for the maintenance of self-renewing and pluripotency in stem cells [66–69]. These results demonstrated that differentially expressed TFs in hESC lines are involved in regulating cell developmental process while expression of non-DETFs mostly involved in metabolic process and biosynthesis process.

### Expression variability on signaling networks among hESC lines

**Fig 5:**
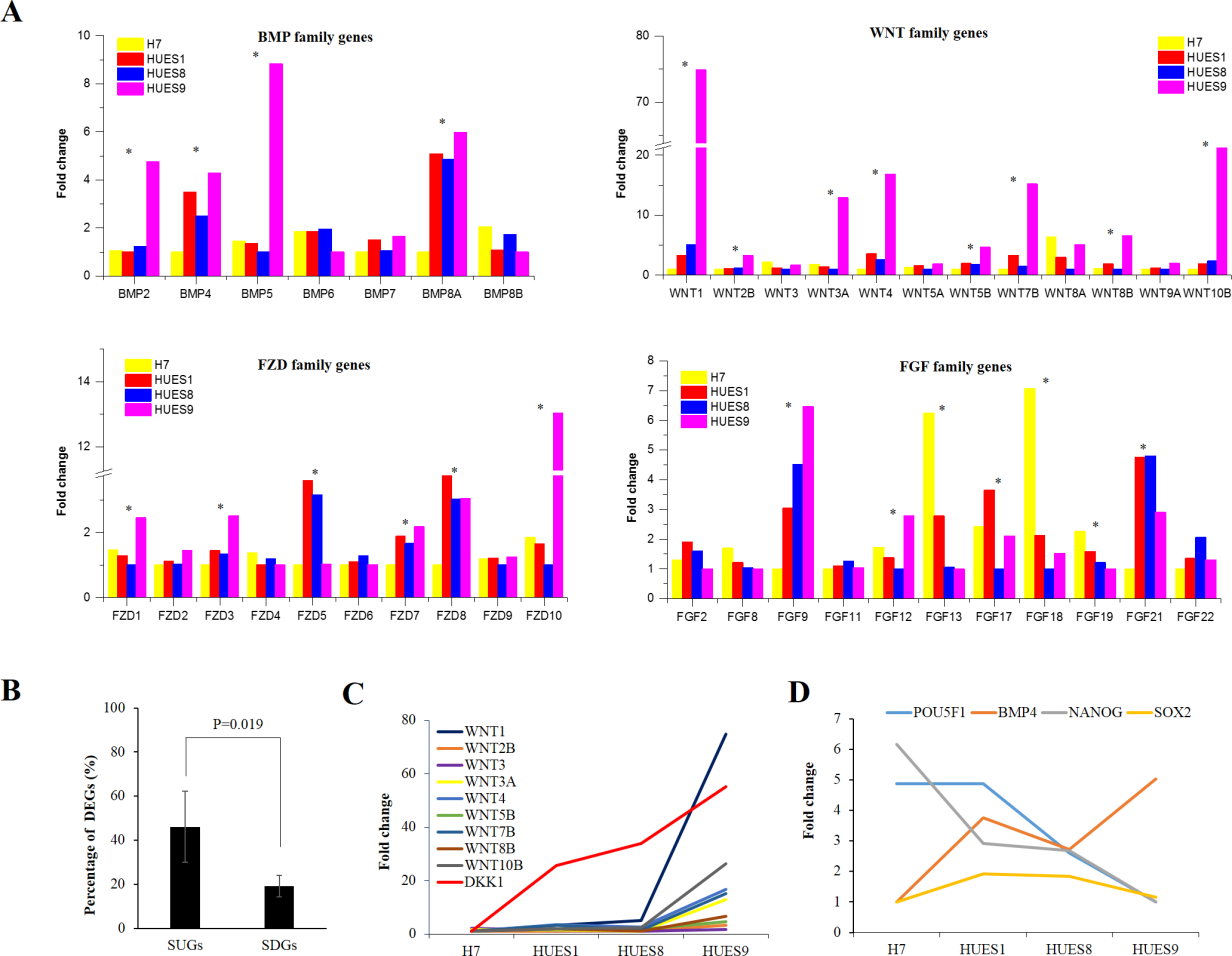
Expression variations of genes involved in IGF/PI3K, FGFMAPK, TGF-β and Wnt signaling pathways in hESC lines H7, HUES1, HUES8 and HUES9. (A) Fold change of BMP, WNT, FDZ and FGF family genes among the four lines. * represents genes differentially expressed at least between two cell lines by FC > 2 and FDR < 0.01. (B) Percentage of DEGs in PUGs and PDGs of the four signaling pathways. Statistically significant were analyzed by student's t-test. (C) Line chart showing fold change of differentially expressed WNT family genes and inhibitor DKK1 in Wnt signaling pathway in the four cell lines. (D) Line chart depicting fold change of BMP4 and pluripotent factors POU5F1 and NANOG in the four cell lines. Fold change of each gene mentioned here was normalized by the minimum CPM of the gene among the four lines.

There are several key signaling pathways required for maintaining pluripotent state while suppressing differentiation in hESCs, including insulin-like growth factor /phosphatidylinositol-3 kinase (IGF/PI3K), fibroblast growth factor (FGF)/Mitogen- activated protein kinase (MAPK), TGF-βand Wnt pathway [7, 70–75]. These finely-balanced and coordinately interacted signaling networks are critical for cell fate decisions in hESCs [73, 76]. Hence, we selected genes of these pathways and investigated their expression variation in the four hESC lines. We totally selected 176 genes involved in these four canonical pathways, in which 60 genes were significantly differentially expressed (S6 Table). Interestingly, many genes we found were ligands or receptors showed great variation among the four cell lines, including bone morphogenetic protein (BMP) genes, wingless-type MMTV integration site family (WNT) genes, fibroblast growth factor (FGF) genes, and frizzled family receptor (FZDR) genes etc. (Fig 5A). Ligands and receptors are upstream signaling inputs, so we wanted to know how their different expression would influence downstream genes expression. We grouped all genes into two classes: signaling upstream genes (SUGs) (including ligands, receptors, and co-receptors) and signaling downstream genes (SDGs) (including kinase, TFs, et.). (S6 Table). Expression variations between SUGs and SDGs were significant different among the four cell lines. 46.07% genes in SUGs were differentially expressed while only 16.11% genes in SDGs showed differential expression (Fig 5B). According to previous studies, whether Wnt pathway is required for human stem cell pluripotency or differentiation are still unclear [7, 76, 77]. Here we found 9 of 12 WNT family genes were differentially expressed, and expression level in HUES9 were much higher than other cell lines. But we noticed that DKK1, one inhibitor of the Wnt signaling pathway, also were significantly upregulated in HUES9 as well (Fig 5C). When we examined the expression level of Wnt family genes between H1 and HUES64 using RNA-seq data downloaded from public database, we can also see that WNT family genes were highly expressed in HUES64 cell line and more than half of the upstream genes (53.85%) in the Wnt signaling pathway presented significantly different expression level (Figure A in S4 Fig). Meanwhile, no significant differences were observed in the expression level of most downstream genes, except LEF1 and APC2 (Figure B in S4 Fig). These results together implicated that hESCs coordinate signal networks to keep the expression of SDGs within a narrow range for the maintenance of pluripotency and self-renew, although the signaling inputs are greatly varied among different hESC lines that could influence their consequent differentiation bias. However, a general mechanism by which differentially expressed genes (such as WNT family genes) between human pluripotent cell lines that precisely coordinated to keep each pluripotent status remains to be elucidated.

## Discussion

Previous studies have confirmed that physiological and functional variability exists among hESC lines, although they have the common properties in the ability to differentiate into any cell type of the body and self-renew indefinitely *in vitro* [24, 38]. One of these variations is that they exhibited different capability to form certain cell type, which could influence their future application [20, 21]. The gene expression profile of hESCs has been explored by several techniques, including serial analysis of gene expression (SAGE), expressed sequence tag (EST) enumeration, microarray analysis and massively parallel signature sequencing [24]. However, most of these studies have been undertaken to unravel the key genes that characterize the status of ‘stemness’, regulate pluripotency and maintain the undifferentiated state. And several other studies were interesting in the comparison of ES cells and iPS cells [24, 38]. Thus, the influence of gene expression variations between hESC lines on their differentiation behavior has yet to be elucidated.

In this work, we wanted to know whether transcriptome variations among hESC lines link to their differentiation bias *in vitro* by RNA-seq analysis. We compared expression profiles of four hESC lines H7, HUES1, HUES8 and HUES9, and totally identified 19, 429 expressed genes, among which 4, 302 (22.14%) genes, including 362 (1.86%) transcription factors, were differentially expressed at least in two cell lines. Functional annotation demonstrated that these DEGs were significantly enriched in developmental processes, such as ectoderm development, mesoderm development (Fig 2B). During the stem cell specification, one cell type commitment accompanied with changes in expression pattern and regulation network, and these changes could potentially function to antagonize other cell types formation. That is to say that differentiation process is a one-or-the-other process. Among the four lines, HUES1, HUES8 and HUES9 have distinct differentiation propensity reported in previous study. Specifically, HUES1 and HUES8 exhibited a tendency to turn on genes characteristic of meso-, endo- and epidermal (skin) lineages, whereas HUES9 showed inclination to ectodermal and neuronal genes [20]. Here, we found that gene expression pattern of HUES1 was more similar to HUES8 than to HUES9 (Fig 3B and S3 Fig). These DEGs upregulated in HUES9 were enriched in nervous system development and ectoderm development, implicating its differentiation direction bias. Accordingly, many genes function in nervous system development and ectoderm development were downregulated in HUES1 and HUES8 comparing to HUES9, meaning less possibility to antagonize endoderm formation. Not surprisingly, upregulated genes in HUES1 and HUES8 showed function enrichment in endoderm development (Fig 3C and 3D). These results indicating their differentiation propensity are in line with previous report [20]. Besides, we compared PAX6 and Nestin expression in spontaneously differentiating embryoid bodies derived from the four cell lines at day 28 by RT-PCR. Results also showed that the level of PAX6 and Nestin expression was significantly higher in HUES 9 than in HUES 1 and HUES 8 (Figure A in S5 Fig).

Transcription factors, especially master transcription factors in the core regulation circuit, such as POU1F5, NANOG, and SOX2, play pivotal role in genes expression regulation to sustain self-renewal and pluripotent state in hESCs, while inhibiting differentiation [19]. However, it has been demonstrated that they also play important role in early mouse and human embryonic cell fate decision [78, 79]. Moreover, self-renewal and distinct lineage specification are orchestrated in hESCs through cross-talk between these pluripotency factors and signal pathways [80–82]. POU5F1 and NANOG specially antagonize neuroectodermal induction, whereas SOX2 is required in this layers. Conversely, POU5F1 and NANOG promote meso and/or endodermal differentiation while SOX2 potently suppress mesendodermal formation [78, 81]. In addition, high levels of POU5F1 enable self-renewal in the absence of BMP4 but specify mesendoderm in the presence of BMP4 while low levels of OCT4 induce embryonic ectoderm differentiation in the absence of BMP4 but specify extraembryonic lineages in the presence of BMP4 [64]. In this study, POU5F1 and NANOG were differentially expressed while SOX2 showed similar expression level in the four lines (Fig 5D). Expression of POU5F1 and NANOG were much higher in HUES1 and HUES8 than in HUES9, and the expression difference were in line with their differentiation bias. H7 had high POU5F1 expression and low BMP4 expression, consistent with previous report [64]. Notably, expression of POU5F1 in HUES9 are lowest and BMP4 are highest compared to other lines. gene expression level of POU5F1 in HUES9 were the lowest and BMP4 were the highest among these four cell lines. But POU5F1 remain present high expression level in POU5F1 and are significantly higher than differentiated EB cells (Fig 4B). Therefore, high levels of POU5F1 together with BMP4 could specify mesendoderm, implicating an antagonistic mechanism in HUES9 whose upregulated genes are significantly enriched in ectoderm development.

BMP family and Wnt family genes play important role in developmental processes. Their temporal and spatial regulation of signals are crucial for special tissue development, such as heart [36]. Although BMP family and Wnt family genes have significant changes in HUES9 cell lines compared to the other three cell lines (Fig 5A), results of direct neural differentiation exhibited that percentage of PAX6^+^ cells derived from these four cell lines were comparative, and efficiency are very high, at about 97% (Figure B and C in S5 Fig). On the other side, when directing the four cell lines to form cardiomyocytes, efficiency was significantly different among them. Specifically, percentage of TNNT2^+^ cells derived from HUES8 and HUES1 are significantly higher than cells derived from HUES9 (P-value = 0.003 by one-way ANOVA) (Figure B in S6 Fig). Contracting cells from HUES9 mainly appeared in large cell clumps (S1–S4Videos). These results indicated that different gene expression patterns in different hESC lines could appreciably impact on target type cells differentiation efficiency, however, differentiation bias could be overcome by finding appropriate direct differentiation methods [24].

In summary, our study demonstrated that DEGs among hESC lines are significantly enriched in developmental processes, involving in ectoderm, mesoderm and endoderm development. Human embryonic stem cells could potentially coordinate genes expression to balance core regulation circuit and maintain un-differentiation state, in which cross-talk between genes, including pluripotency factors and genes participating in signaling transduction, were involved. Some of these genes could affect their differentiation behavior, but they collectively keep hESC in a stable status. When the balance was broken, expression variations between lines eventually contribute to their differentiation propensity *in vitro*. The degree to which these differentially expressed genes contribute to the capability of hESCs forming a certain cell type, and whether some of these DEGs have larger weight than others when used as markers to predict their differentiation behavior remain to be determined. More data of gene expression and efficiency in forming desired cell types from different hESC lines are needed. Furthermore, the underlying molecular mechanisms by which DEGs affect differentiation bias and whether recently constructed naïve hESC lines [83–86] are more homogeneous than conventional cell lines need to be investigated.

## Supporting information

S1 Fig Morphology of the four hESC lines H7(top left), HUES1 (top right), HUES8 (bottom left) and HUES9 cultured in feeder-free medium. Bar, 100 μm.
(TIF)

S2 Fig Differential expression analysis in hESC lines H7, HUES1, HUES8 and HUES9. (A) GO-slim biological process enrichment analysis of DEGs between H7 and HUES1. (B) GO-slim biological process enrichment analysis of DEGs between H7 and HUES8. (C) GO-slim biological process enrichment analysis of DEGs between H7 and HUES9. (D) GO-slim biological process enrichment analysis of DEGs between H1 and HUES64 downloaded from public available RNA-seq data.
(TIF)

S3 Fig Differential expression analysis by two-two comparison. (A) H7 compared to HUES1, HUES8 and HUES9. (B) HUES1 compared to H7, HUES8 and HUES9. (C) HUES8 compared to H7, HUES1 and HUES9. (D) HUES9 compared to H7, HUES1 and HUES8. Upregulated: logFC > 1 and FDR < 0.01, downregulated: logFC <_ -1 and FDR < 0.01.
(TIF)

S4 Fig Comparison of expression level of Wnt signaling pathway genes between hESC lines HUES64 and H1. (A) Expression variations of genes in Wnt signaling pathway upstream component between hESC lines HUES1 and H1. (B) Expression variations of genes in Wnt signaling pathway downstream component between hESC lines HUES1 and H1.
(TIF)

S5 Fig Neural differentiation from H7, HUES1, HUES8 and HUES9. (A) Fold change of PAX6 and Nestin expression in spontaneously differentiating embryoid bodies derived from H7, HUES1, HUES8 and HUES9 at day 28. (B) Percentage of PAX6^+^ cells derived from H7, HUES1, HUES8 and HUES9. (C) Example of flow cytometry analysis for PAX6^+^ cells derived from H7, HUES1, HUES8 and HUES9.
(TIF)

S6 Fig Cardiac differentiation from H7, HUES1, HUES8 and HUES9. (A) Example of cardiomyocytes morphology in culture derived from H7, HUES1, HUES8 and HUES9. (B) Percentage of TNNT2^+^ cells derived from H7, HUES1, HUES8 and HUES9. (C) Example of flow cytometry analysis for TNNT2^+^ cells derived from H7, HUES1, HUES8 and HUES9.
(TIF)

S1 Table List of genes expressed in the four hESC lines. (XLSX)

S2 Table List of top 1000 highly expressed genes in the four hESC lines. (XLSX)

S3 Table Different expression genes in the four hESC lines. (XLSX)

S4 Table DEGs from two-two cell lines comparisons. (XLSX)

S5 Table Transcript factor genes expressed in the four hESC lines. (XLSX)

S6 Table Signaling pathway genes expressed in the four hESC lines. (XLSX)

S7 Table Results of GO biological process complete enrichment analysis for upregulated genes in HUES1 and HUES8 compared to HUES9. (XLSX)

S1 Video Example of cardiomyocyte contracting derived from H7. (MP4)

S2 Video Example of cardiomyocyte contracting derived from HUES1. (MP4)

S3 Video Example of cardiomyocyte contracting derived from HUES8. (MP4)

S4 Video Example of cardiomyocyte contracting derived from HUES9. (MP4)
