# Supplementary figures and images for: Transcriptome variations among human embryonic stem cell lines are associated with their differentiation propensity
Source: PLoS One. 2018 Feb 14;13(2):e0192625. doi: 10.1371/journal.pone.0192625 (PMC5812638; doi:10.1371/journal.pone.0192625)

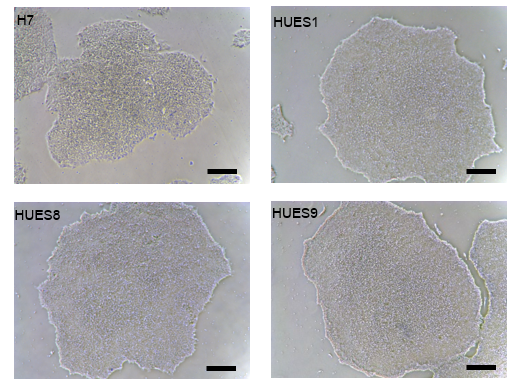

Supplement: S1 Fig — Bar, 100 μm. (TIF) [file pone.0192625.s001.tif]

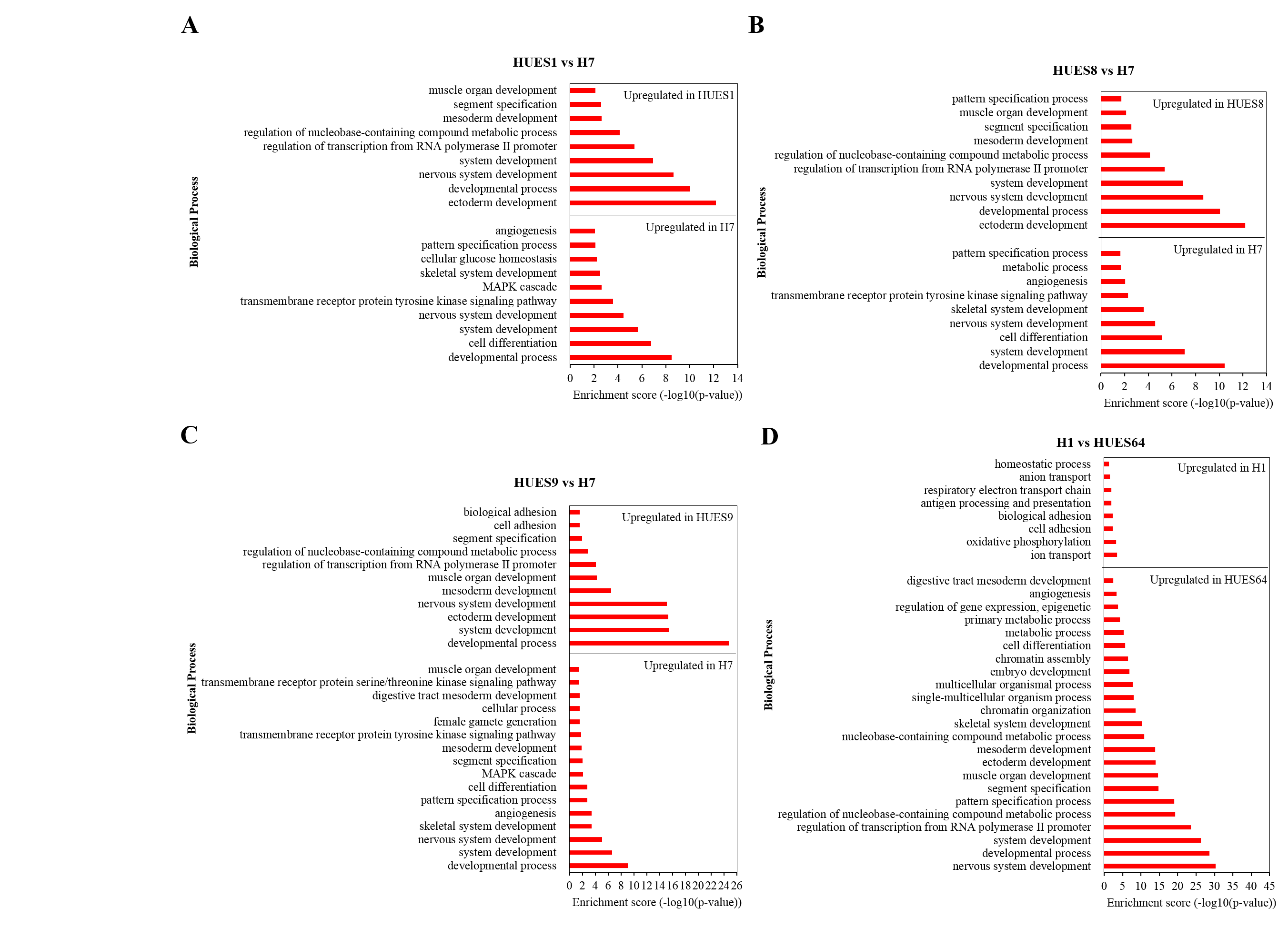

Supplement: S2 Fig — (A) GO-slim biological process enrichment analysis of DEGs between H7 and HUES1. (B) GO-slim biological process enrichment analysis of DEGs between H7 and HUES8. (C) GO-slim biological process enrichment analysis of DEGs between H7 and HUES9. (D) GO-slim biological process enrichment analysis of DEGs between H1 and HUES64 downloaded from public available RNA-seq data. (TIF) [file pone.0192625.s002.tif]

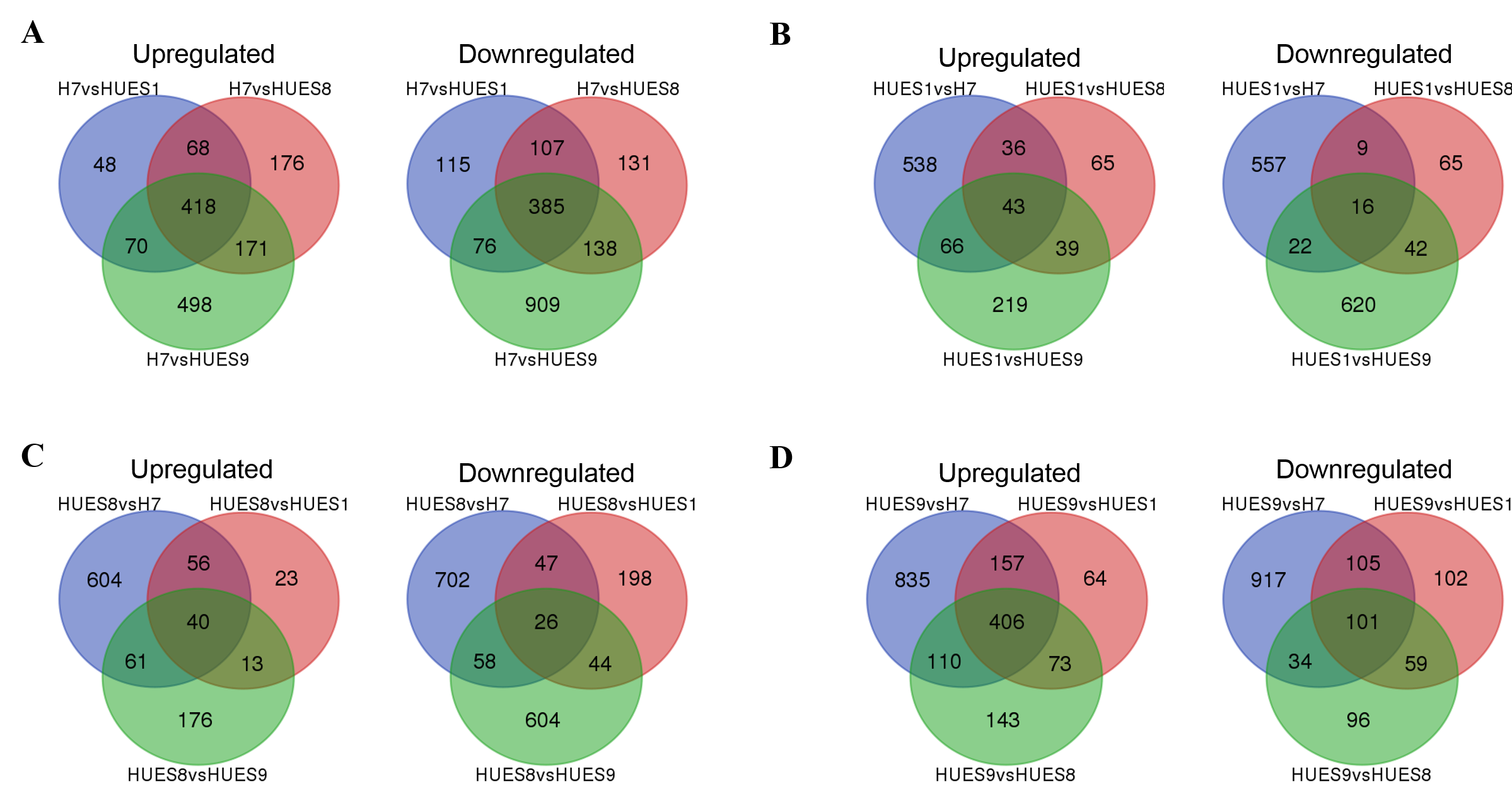

Supplement: S3 Fig — (A) H7 compared to HUES1, HUES8 and HUES9. (B) HUES1 compared to H7, HUES8 and HUES9. (C) HUES8 compared to H7, HUES1 and HUES9. (D) HUES9 compared to H7, HUES1 and HUES8. Upregulated: logFC > 1 and FDR < 0.01, downregulated: logFC <_ -1 and FDR < 0.01. (TIF) [file pone.0192625.s003.tif]

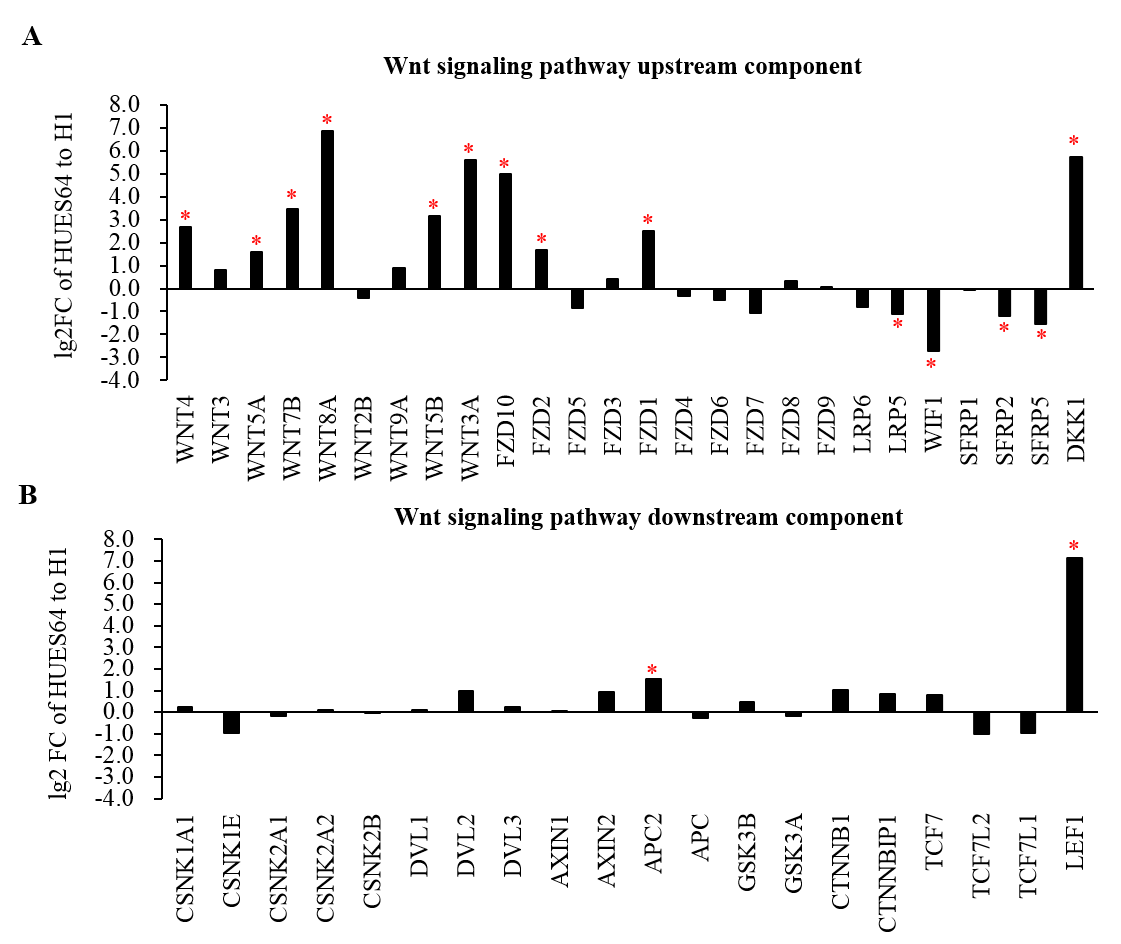

Supplement: S4 Fig — (A) Expression variations of genes in Wnt signaling pathway upstream component between hESC lines HUES1 and H1. (B) Expression variations of genes in Wnt signaling pathway downstream component between hESC lines HUES1 and H1. (TIF) [file pone.0192625.s004.tif]

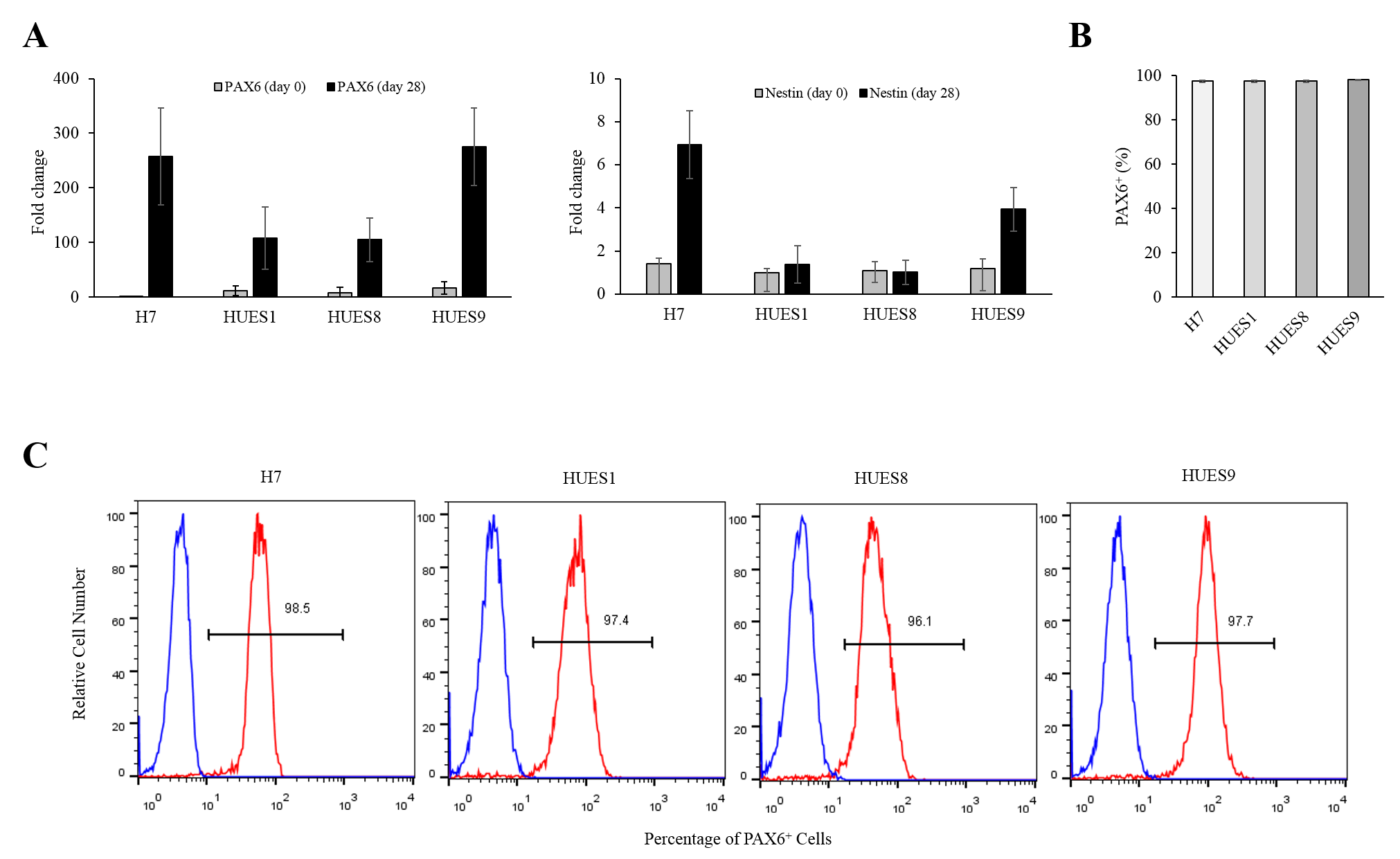

Supplement: S5 Fig — (A) Fold change of PAX6 and Nestin expression in spontaneously differentiating embryoid bodies derived from H7, HUES1, HUES8 and HUES9 at day 28. (B) Percentage of PAX6+ cells derived from H7, HUES1, HUES8 and HUES9. (C) Example of flow cytometry analysis for PAX6+ cells derived from H7, HUES1, HUES8 and HUES9. (TIF) [file pone.0192625.s005.tif]

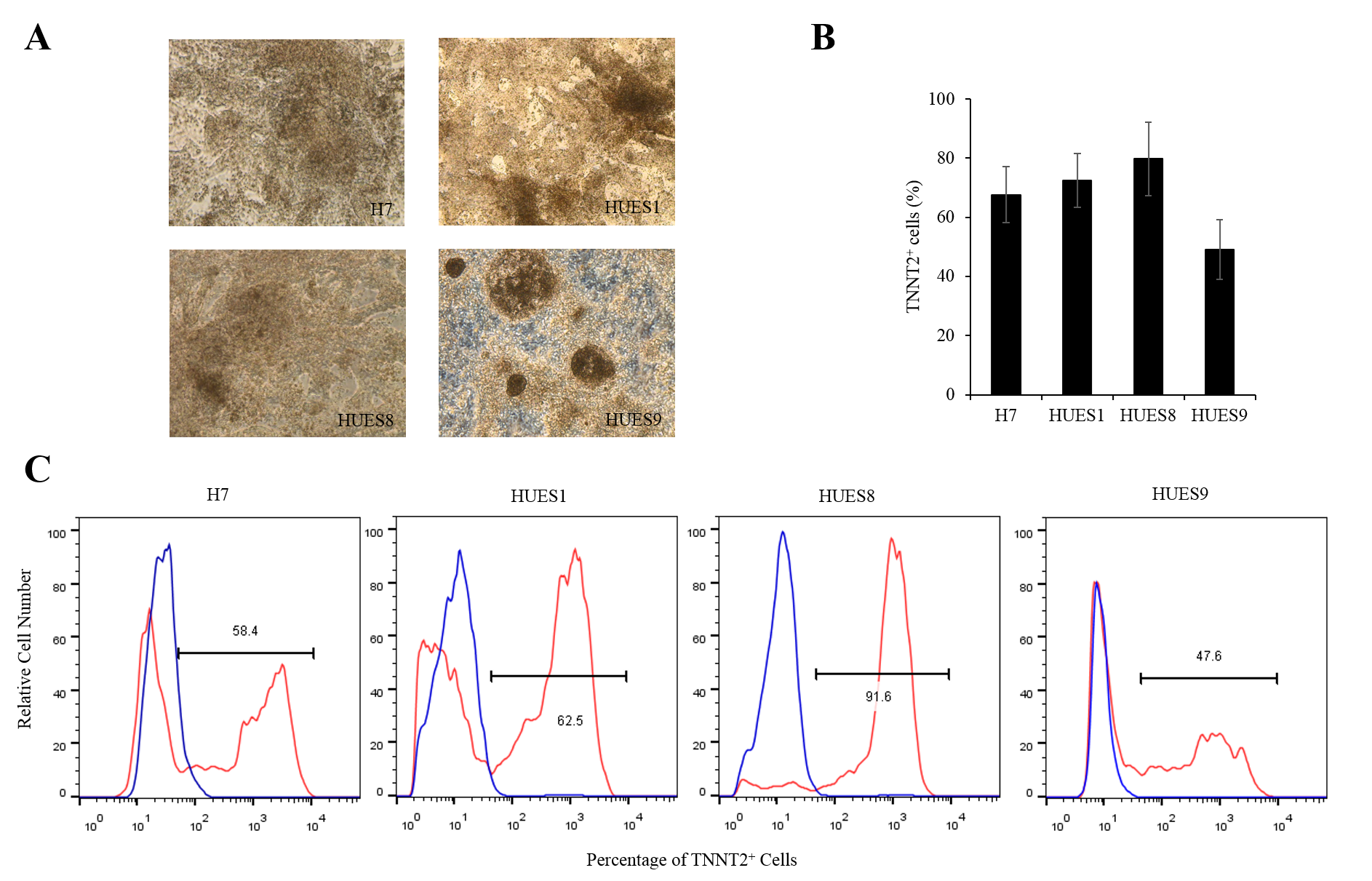

Supplement: S6 Fig — (A) Example of cardiomyocytes morphology in culture derived from H7, HUES1, HUES8 and HUES9. (B) Percentage of TNNT2+ cells derived from H7, HUES1, HUES8 and HUES9. (C) Example of flow cytometry analysis for TNNT2+ cells derived from H7, HUES1, HUES8 and HUES9. (TIF) [file pone.0192625.s006.tif]
